# Heart and Brain Tissue Banks for Research on Co-Occurring Cardiovascular and Neurological/Psychiatric Disorders

**DOI:** 10.1155/2009/427840

**Published:** 2009-04-15

**Authors:** Milos D. Ikonomovic

**Affiliations:** Departments of Neurology and Psychiatry, University of Pittsburgh School of Medicine, Pittsburgh, PA 15261, USA

## Abstract

Epidemiological studies point to a strong and possibly causal association of psychiatric and neurological disorders with cardiovascular disease (CVD). Mechanistic links between these co-occurring illnesses are not well understood. Better insight into their relationship could help identify novel diagnostic markers and therapeutic targets. For successful translation of basic biomedical research into clinical practice, analyses of postmortem human tissues are essential. However, current tissue banks dedicated to psychiatric and neurological research collect only brain tissue samples deemed most important to the institution's participating investigators. While this practice is often dictated by budget constraints, restricted tissue storage space and other practical reasons, it limits the ability of the biological research community to access and study multiple organ systems relevant to cardiovascular and neuronal systems dysfunction. This problem is worsened when clinical records pertaining to coexistent systemic pathology are not available. To promote further understanding of co-occurring CVD and psychiatric/neurological disorders, efforts should be made to support tissue banks that harvest heart, coronary arteries, and aorta samples as well as brain tissue, from the same subjects.

A growing awareness of the links between diseases of the cardiovascular system and pathologies of the central nervous system, including psychiatric and neurological disorders, has promulgated interest in understanding the pathophysiological mechanisms underlying these associations [1]. It is apparent that studying the brain in isolation hampers understanding the etiology of the most common neurodegenerative diseases (such as Alzheimer's disease; AD). This view has led to reassessment of our current methods of AD research and increased emphasis on more thorough evaluation of the risk factors and pathophysiological processes in multiple organ systems [2].

Extensive efforts have been made to elucidate the role of elevated serum cholesterol, APOE4 genotype, heart disease, and cerebral hypoperfusion in the pathogenesis of AD [3]; however only a few studies have been able to examine possible correlations between hallmark lesions of AD and measures of CVD pathology in the same subjects postmortem. The latter approach has resulted in some intriguing observations. For example, Beeri and colleagues reported that indices of coronary artery disease, assessed histologically postmortem, correlated directly with densities of neuritic plaques in the hippocampus, entorhinal cortex, and neocortex [4]. These results supported previous findings of a link between AD pathology and coronary artery disease [5]; but they were not consistent with some other studies that sought to correlate AD lesions with the extent of CVD at autopsy [6, 7]. To resolve the controversy, additional studies utilizing brain, heart, and artery tissues from the same subject are critical. This could provide new insight into possible links between cardiovascular and neurodegenerative diseases, especially when medical records are incomplete and detailed antemortem assessments of cardiovascular function (e.g., coronary angiography, electrocardiography) were not performed in psychiatric and neurological patients who came to autopsy.

A prominent example of comorbid psychiatric and cardiovascular disorders is the association between depression and coronary heart disease (CHD). Depression is associated with increased cardiovascular morbidity and mortality [8] and with prominent increases in cardiovascular health care costs [9]. Despite recent recommendations that all patients with CHD be screened for depression [8], additional research is needed to determine whether existing depression treatments affect cardiac outcomes [10]. Hence, better understanding of biological mechanisms linking CHD and depression is needed to direct clinical trials, for example, to select the most appropriate therapeutic targets and treatment modalities for these comorbid disorders.

It is conceivable that studies using postmortem brain and heart/vascular tissues from the same subject would be advantageous to the cardiovascular psychiatry/neurology research; however such samples are not harvested together routinely from autopsies of normal and diseased adults. Considering the complexity of current brain banking efforts [11, 12], it is not surprising that tissue banks of multiple-organ autopsy samples from psychiatric and neurological patients are not readily available. Nevertheless, banking protocols can be modified once the need for harvesting additional tissues has been identified. For example, the observation that retinal ganglion cells and their axons are affected in AD has prompted some Alzheimer's Disease Research Center (ADRC) brain banks to start collecting autopsy samples of retinas and optic nerve. Larger brain banks that support investigations into specific neurodegenerative disorders, particularly those banks structured within the ADRC or other large National Institutes of Health-funded initiatives for longitudinal studies of the elderly and AD, have the advantage of being able to obtain brain and spinal cord autopsy samples, the subject's blood and cerebrospinal fluid samples, and detailed antemortem clinical and imaging data. Ideally, banking efforts would be extended to include additional organs and tissues, such as the heart and blood vessels, to their specimens. The Brain Bank of the Brazilian Aging Brain Study Group has undertaken such an ambitious multiorgan banking project. This tissue bank has collected more than 1600 brains and has implemented an autopsy protocol for harvesting additional organs/tissues such as heart, cervical carotids, and kidneys [13]. Carotid, coronary, and Willis' Circle arteries are evaluated for atherosclerosis, and heart valves and ventricular muscle are examined for chronic effects of hypertension. To establish successful tissue banking resources of such magnitude, great dedication and effort will be needed from researchers as well as willing donors. Some of the cohorts that would be particularly valuable for planning the brain-heart donation protocol include participants in prospective studies of CVD and dementia [14].

Several issues should be considered before initiating the extension of an existing brain bank into a multiorgan bank. First, a detailed and comprehensive protocol would have to be formulated to ensure standardized sampling procedures from multiple organs. Next, a dedicated and adequate source of funding must be secured; tight budgets combined with insufficient personnel and tissue storage capacities of most brain banks in the USA and abroad restrict large scale storage of harvested tissues. Finally, greater efforts must be made to promote collaborations of basic and clinical neuroscientists with experts in CVD. These collaborations are invaluable in the light of the co-occurrence of cardiovascular and psychiatric/neurological disorders as well as the progressive multisystem dysfunction that accompanies cognitive decline in many elderly individuals. By expanding our existing brain tissue banks to accommodate the emerging need for a multiorgan/tissue oriented research, multidisciplinary collaborations would be fostered to provide broader insight into the disease process. This could be achieved by requesting improved funding for such initiatives, along with matching education and outreach efforts to ensure greater public support for such projects.
